# Supplementary material for: Human DNA2 possesses a cryptic DNA unwinding activity that functionally integrates with BLM or WRN helicases
Source: eLife. 2016 Sep 9;5:e18574. doi: 10.7554/eLife.18574 (PMC5030094; doi:10.7554/eLife.18574)
Supplement: Supplementary file 1. — (A) Codon-optimized nucleotide sequence of hDNA2 gene for the expression in Sf9 cells. (B) Sequences of oligonucleotides used in this study. (C) Oligonucleotide-based DNA substrates used in this study. DOI: http://dx.doi.org/10.7554/eLife.18574.016 [file elife-18574-supp1.doc]

| **Supplementary file 1A: Codon-optimized nucleotide sequence of hDNA2 gene for the expression in *Sf9* cells** |
| --- |
| ATGGAGCAATTGAACGAACTCGAACTGTTGATGGAAAAGTCATTCTGGGAGGAGGCTGAACTGCCCGCCGAACTCTTCCAAAAGAAGGTGGTCGCTTCGTTCCCAAGGACTGTCCTGTCCACAGGAATGGACAACAGATACCTCGTGTTGGCCGTCAACACCGTTCAGAACAAGGAGGGTAACTGCGAAAAGAGGCTCGTGATCACTGCTTCACAATCGTTGGAGAACAAGGAATTGTGCATCCTGAGAAACGACTGGTGTTCAGTTCCCGTGGAGCCTGGCGATATCATCCACCTGGAAGGAGACTGCACCTCCGATACTTGGATCATCGACAAGGATTTCGGATACCTGATCCTCTACCCTGACATGCTGATCTCTGGTACTAGCATCGCTTCCTCTATCCGTTGTATGCGTCGCGCCGTCCTCTCCGAGACATTCCGTAGCTCAGACCCCGCCACTCGCCAGATGTTGATCGGCACAGTGCTGCACGAAGTCTTCCAAAAGGCTATCAACAACTCTTTCGCCCCTGAGAAGCTCCAGGAATTGGCTTTCCAGACTATCCAAGAGATCAGGCACCTGAAGGAAATGTACAGATTGAACCTGTCCCAGGACGAGATCAAGCAAGAGGTGGAAGATTACCTGCCCTCTTTCTGCAAGTGGGCTGGAGACTTCATGCACAAGAACACTAGCACAGATTTCCCCCAGATGCAACTCTCATTGCCTTCGGACAACTCCAAGGATAACTCTACCTGTAACATCGAGGTTGTGAAGCCCATGGACATCGAGGAATCTATCTGGAGCCCTCGTTTCGGCCTGAAGGGAAAGATCGATGTCACAGTTGGAGTGAAGATCCACCGCGGTTACAAGACCAAGTACAAGATCATGCCACTGGAGCTCAAGACTGGCAAGGAGTCCAACTCTATCGAACACAGGTCTCAGGTCGTTCTGTACACTCTGCTCAGCCAAGAGAGGAGAGCTGACCCCGAAGCCGGCTTGCTGCTCTACCTCAAGACCGGACAGATGTACCCCGTCCCTGCTAACCACCTGGATAAGAGGGAGTTGCTGAAGCTCAGAAACCAGATGGCCTTCTCTTTGTTCCACCGTATCAGCAAGTCAGCTACCCGCCAGAAGACTCAACTGGCCAGCCTCCCTCAGATCATCGAGGAAGAGAAGACTTGCAAGTACTGTTCACAAATCGGCAACTGCGCTCTGTACTCGCGCGCCGTGGAGCAGCAAATGGACTGTTCGTCCGTGCCCATCGTCATGCTCCCTAAGATCGAAGAGGAAACACAGCACCTCAAGCAAACCCACTTGGAGTACTTCTCTCTGTGGTGCTTGATGCTGACCCTCGAATCGCAGTCCAAGGACAACAAGAAGAACCACCAAAACATCTGGCTGATGCCTGCTTCCGAGATGGAAAAGTCTGGAAGCTGCATCGGTAACCTCATCCGCATGGAGCACGTTAAGATCGTGTGCGACGGCCAGTACCTGCACAACTTCCAGTGTAAGCACGGCGCTATCCCAGTGACCAACCTCATGGCCGGTGACAGGGTCATCGTTTCGGGCGAGGAAAGGTCCTTGTTCGCCCTGTCTAGAGGTTACGTCAAGGAGATCAACATGACCACTGTTACTTGCCTCTTGGACAGGAACCTCAGCGTCTTGCCCGAATCAACACTGTTCAGACTCGATCAGGAGGAAAAGAACTGTGACATCGATACACCTTTGGGCAACCTGTCCAAGCTCATGGAGAACACCTTCGTGTCTAAGAAGTTGCGTGACCTGATCATCGATTTCCGCGAACCACAGTTCATCTCCTACTTGTCTAGCGTTCTGCCGCACGACGCTAAGGATACCGTGGCCTGCATCCTCAAGGGTTTGAACAAGCCACAGAGGCAAGCCATGAAGAAGGTCCTGCTCAGCAAGGACTACACCCTGATCGTTGGCATGCCGGGAACTGGCAAGACAACCACTATCTGCACACTCGTGAGAATCTTGTACGCTTGTGGATTCTCCGTCTTGCTGACATCCTACACCCACTCTGCTGTTGACAACATCCTCTTGAAGCTGGCCAAGTTCAAGATCGGCTTCTTGCGTCTGGGACAGATCCAAAAGGTGCACCCCGCTATCCAGCAATTCACCGAGCAGGAAATCTGCCGCTCAAAGTCGATCAAGTCGCTGGCTCTGCTCGAGGAACTCTACAACTCCCAGTTGATCGTCGCCACAACCTGTATGGGAATCAACCACCCTATCTTCTCTCGTAAGATCTTCGACTTCTGCATCGTGGATGAGGCCTCACAGATCTCGCAACCAATCTGTCTGGGCCCGCTCTTCTTCAGCCGTCGCTTCGTGTTGGTCGGAGACCACCAGCAACTGCCCCCTCTCGTCTTGAACCGTGAGGCTCGCGCCCTGGGCATGTCCGAGTCTCTGTTCAAGAGGCTCGAACAGAACAAGTCTGCTGTGGTCCAGCTGACTGTGCAATACAGAATGAACAGCAAGATCATGAGCTTGTCAAACAAGCTGACCTACGAGGGCAAGCTCGAATGCGGCTCAGACAAGGTTGCTAACGCCGTGATCAACTTGCGCCACTTCAAGGATGTCAAGCTGGAGCTCGAATTCTACGCCGACTACTCCGATAACCCCTGGTTGATGGGTGTGTTCGAGCCAAACAACCCGGTCTGCTTCCTGAACACCGACAAGGTCCCAGCTCCGGAGCAGGTTGAAAAGGGTGGCGTCTCAAACGTTACCGAAGCCAAGCTCATCGTTTTCTTGACTTCGATCTTCGTGAAGGCTGGATGCTCGCCATCCGACATCGGTATCATCGCCCCGTACCGTCAGCAACTGAAGATCATCAACGATTTGCTGGCTCGCTCAATCGGTATGGTTGAGGTGAACACCGTGGACAAGTACCAGGGCCGTGATAAGTCCATCGTCCTGGTTAGCTTCGTGCGCTCAAACAAGGACGGTACAGTCGGCGAGCTCTTGAAGGATTGGAGGAGACTGAACGTCGCTATCACCCGTGCCAAGCACAAGCTCATCCTGCTCGGCTGCGTGCCATCCCTGAACTGTTACCCACCGCTCGAGAAGTTGCTGAACCACCTGAACAGCGAAAAGCTGATCATCGACCTCCCGTCACGCGAGCACGAATCCCTCTGCCACATCCTCGGCGACTTCCAGCGTGAATAA |
| **Supplementary file 1A:** 5'-3' nucleotide sequence of human DNA2 gene codon-optimized for the expression in Sf9 insect cells. |

| **Supplementary file 1B:** **Sequences of oligonucleotides used in this study.** | |
| --- | --- |
| **Name** | **Sequence (5' to 3')** |
| Primer 308 | GCTGGTTTAGGACGCACTTG |
| X12-3 | GACGTCATAGACGATTACATTGCTAGGACATGCTGTCTAGAGACTATCGC |
| X12-3 SC | TTGCTAGGACATGCTGTCTAGAGACTATCGC |
| #292 | GGATTACATTGCTAGGACATGCTGTCTAGAGACTATCGC |
| X12-4C | GCGATAGTCTCTAGACAGCATGTCCTAGCAATGTAATCGTCTATGACGTC |
| X12-4SC | GCGATAGTCTCTAGACAGCATGTCCTAGCAA |
| X12-3 HJ1S | AAAAAAAAAAAAAAAAAAATAACCTAGCGATGGAACGTAAGTCGCGAT |
| X12-3 TOPL | GACGTCATAGACGATTACATTGCTAGGACATGCTGTCTAGAGACTATCGCGACTTACGTTCCATCGCTAGGTTATTTTTTTTTTTTTTTTTTT |
| X12-4NC | GCGATAGTCTCTAGACAGCATGTCCTAGCAAGCCAGAATTCGGCAGGCTA |
| X12-3 HJ2Sb | ATTACGATTCGTTACCCATTCACTGTCAGAAGGCACCAGATAGATCTC |
| X12-3 HJ3 | GAGATCTATCTGGTGCCTTCTGACAGTGAATGGGTAACGAATCGTAATAGTCTCTAGACAGCATGTCCTAGCAATGTAATCGTCTATGACGTC |
| **Supplementary file 1B:** Nucleotide sequences of oligonucleotides used in this study. | |

| **Supplementary file 1C: Oligonucleotide-based DNA substrates used in this study.** | | |
| --- | --- | --- |
| **DNA substrate:** | **5' labeled oligonucleotide:** | **Unlabeled oligonucleotide:** |
| ssDNA (20 nt) | Primer 308 | - |
| ssDNA (50 nt) | X12-3 | - |
| dsDNA (31 bp) | X-12-3SC | X12-4SC |
| dsDNA (50 bp) | X12-3 | X12-4C |
| 5’ overhang (19 nt / 31 bp) | X12-3 | X12-4SC |
| 5’ overhang (30 nt / 31 bp) | #292 | X12-4SC |
| 5’ overhang (45 nt / 48 bp) | X12-3 HJ1S | X12-3 TOPL |
| 3’ overhang (19 nt / 31 bp) | X12-3SC | X12-4NC |
| 3’ overhang (45 nt / 48 bp) | X12-3 HJ2Sb | X12-3 HJ3 |
| Y-structure (19 nt /31 bp) | X12-3 | X12-4NC |
| **DNA substrate:** | **3' labeled oligonucleotide:** | **Unlabeled oligonucleotide:** |
| ssDNA (93 nt) | X12-3HJ3 | **-** |
| Y-structure (45 nt / 48 bp) | X12-3HJ3 | X12-3TOPL |
| **Supplementary file 1C:** Oligonucleotide-based DNA substrates used in this study and their components. The following substrates were used for the assays: ssDNA (50 nt), dsDNA (50 bp), 5' overhang (19 nt / 31 bp), 3' overhang (19 nt / 31 bp), Y-structure (19 nt /31 bp), unless indicated differently in the figure legends. | | |
